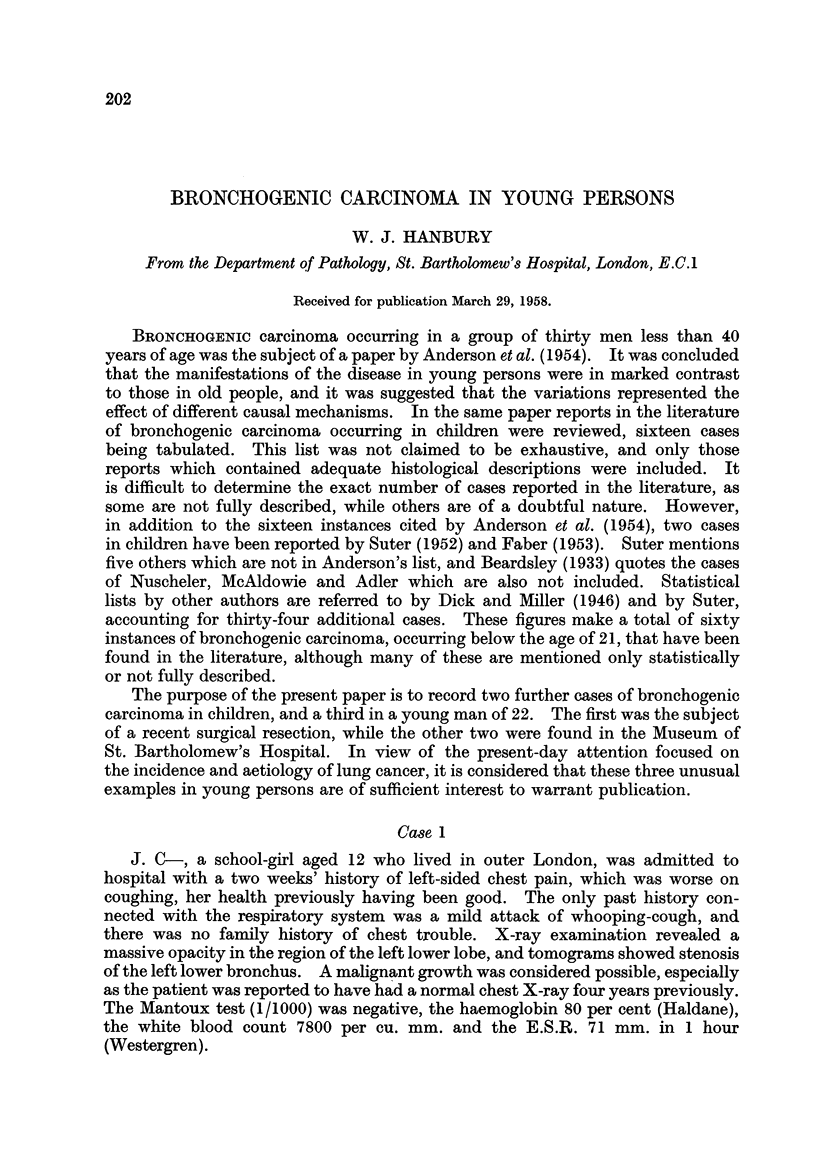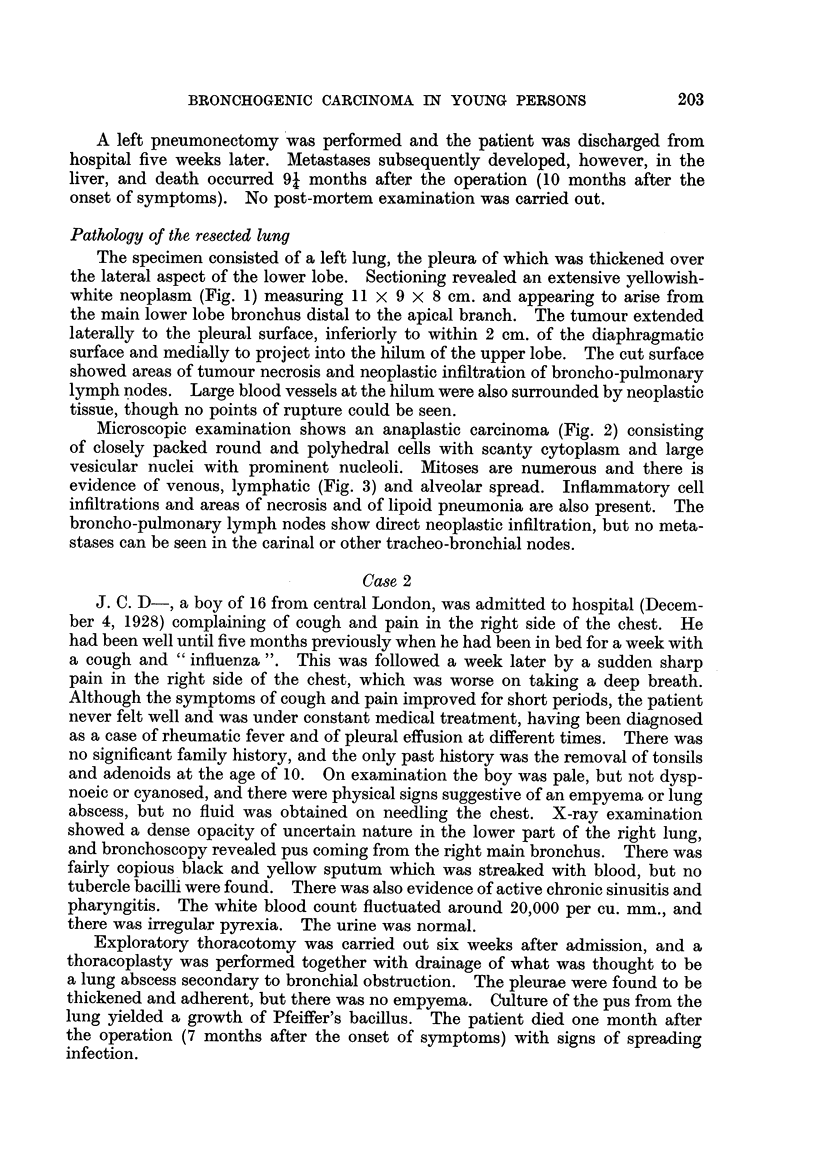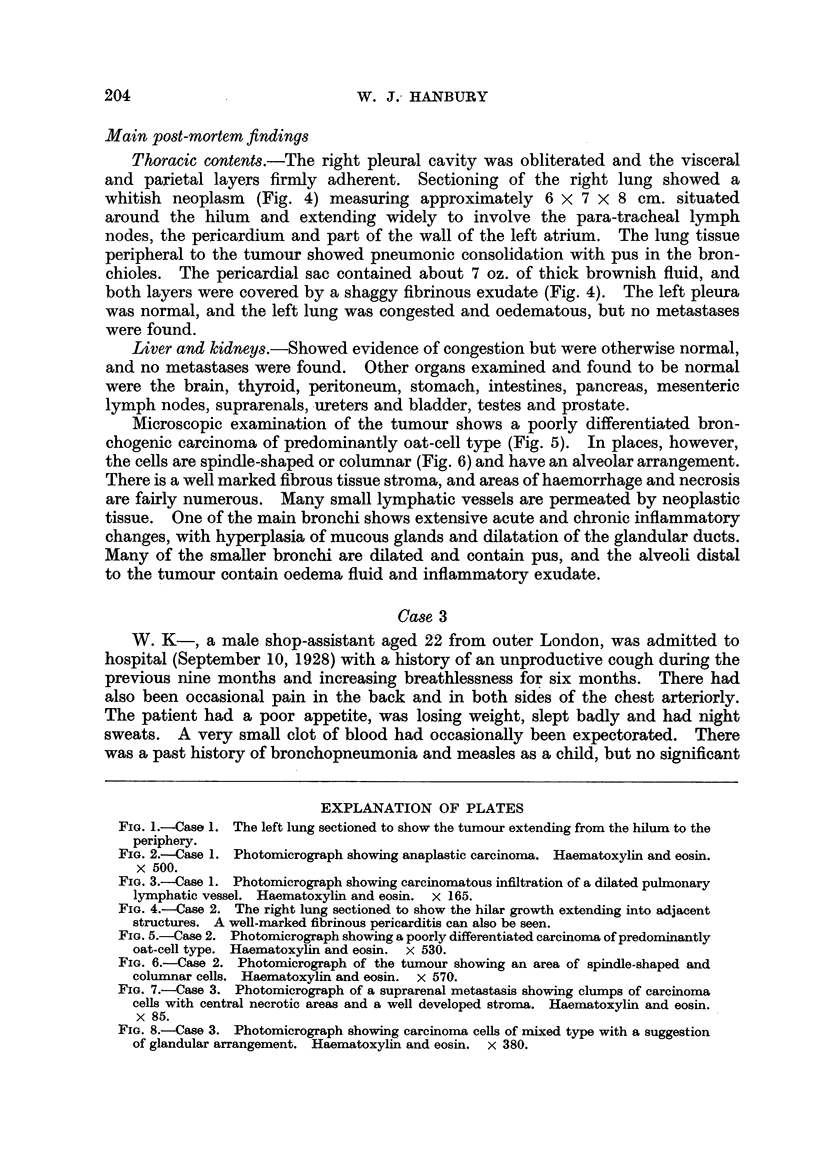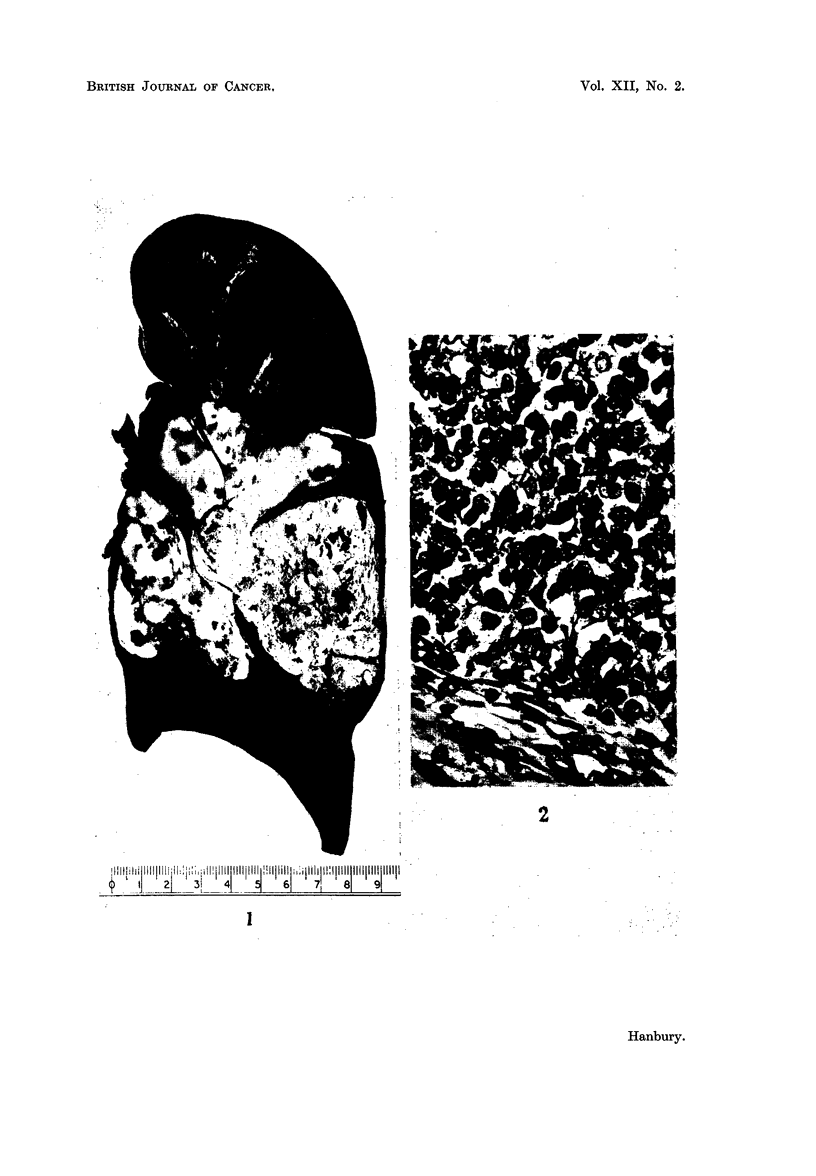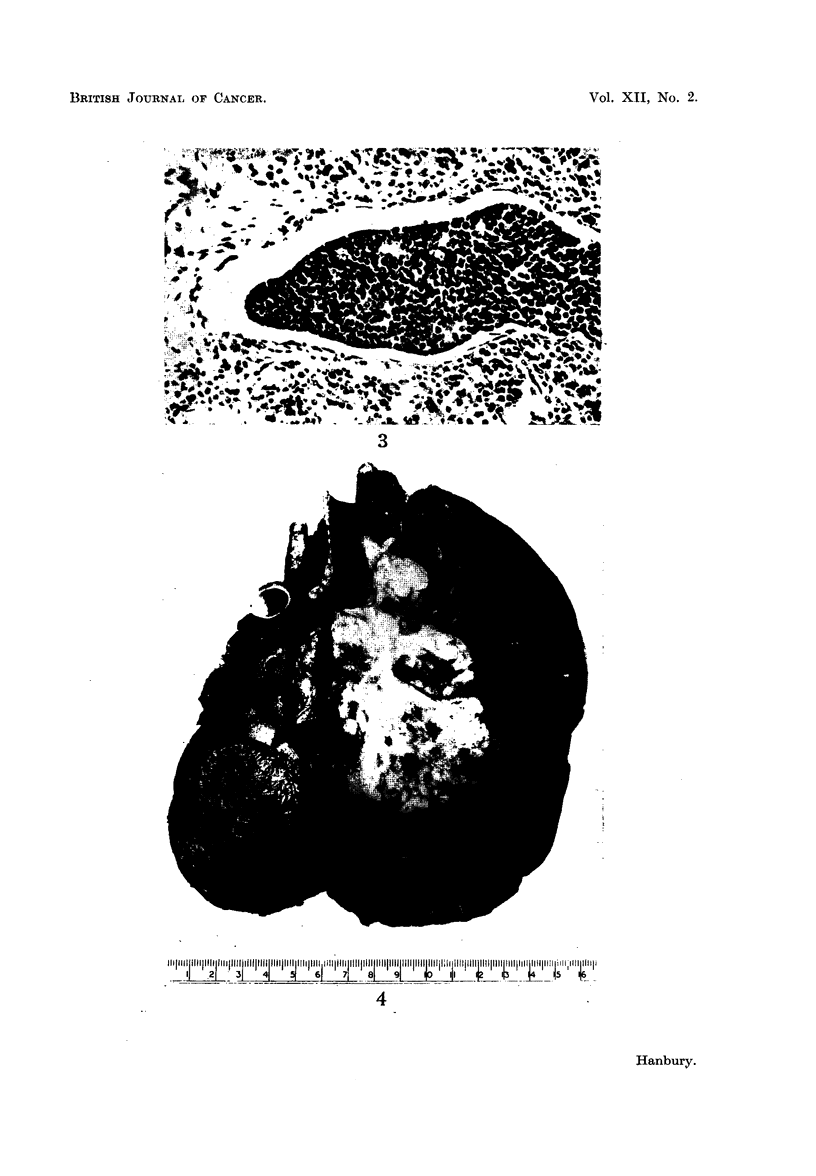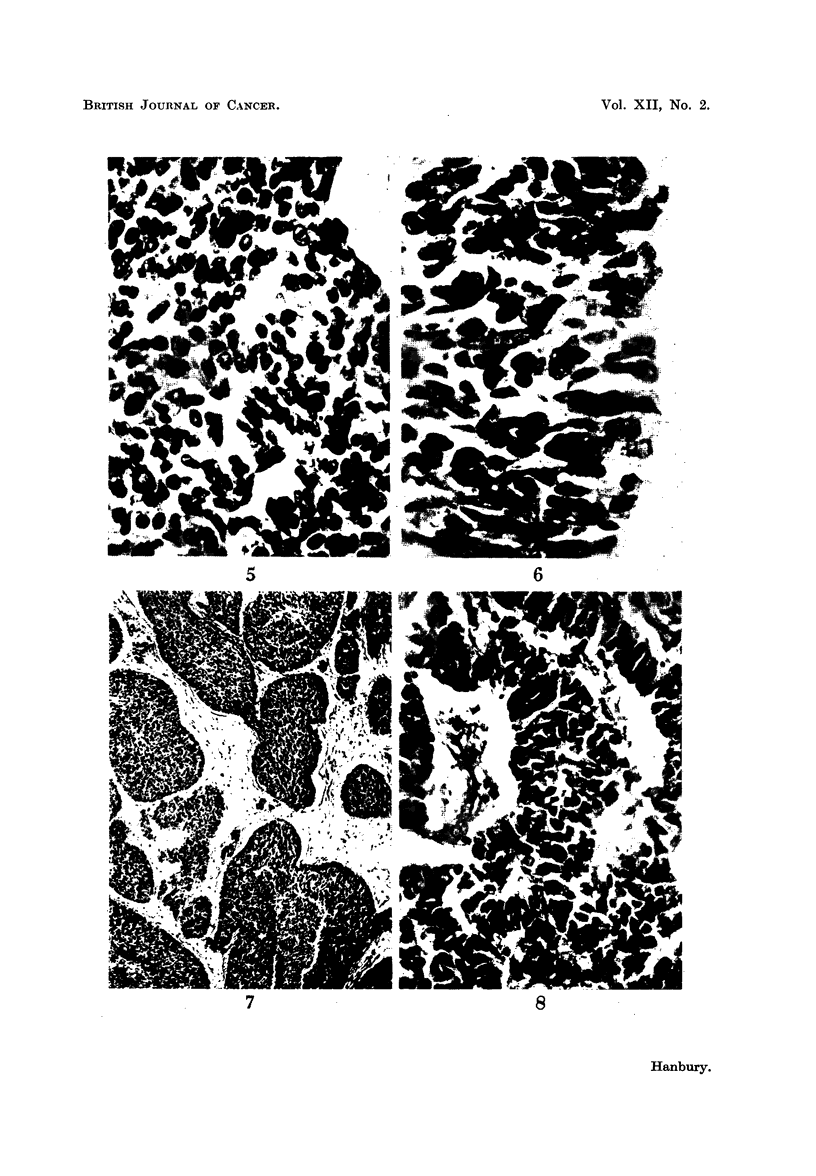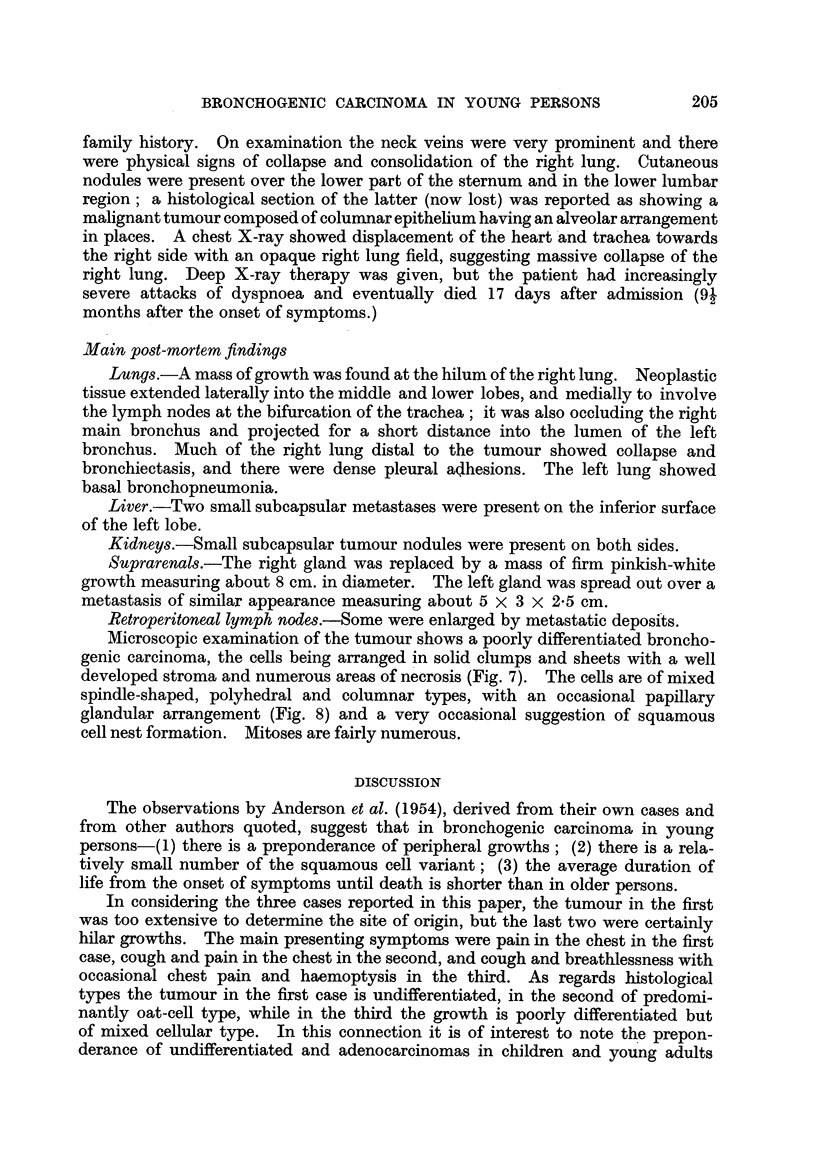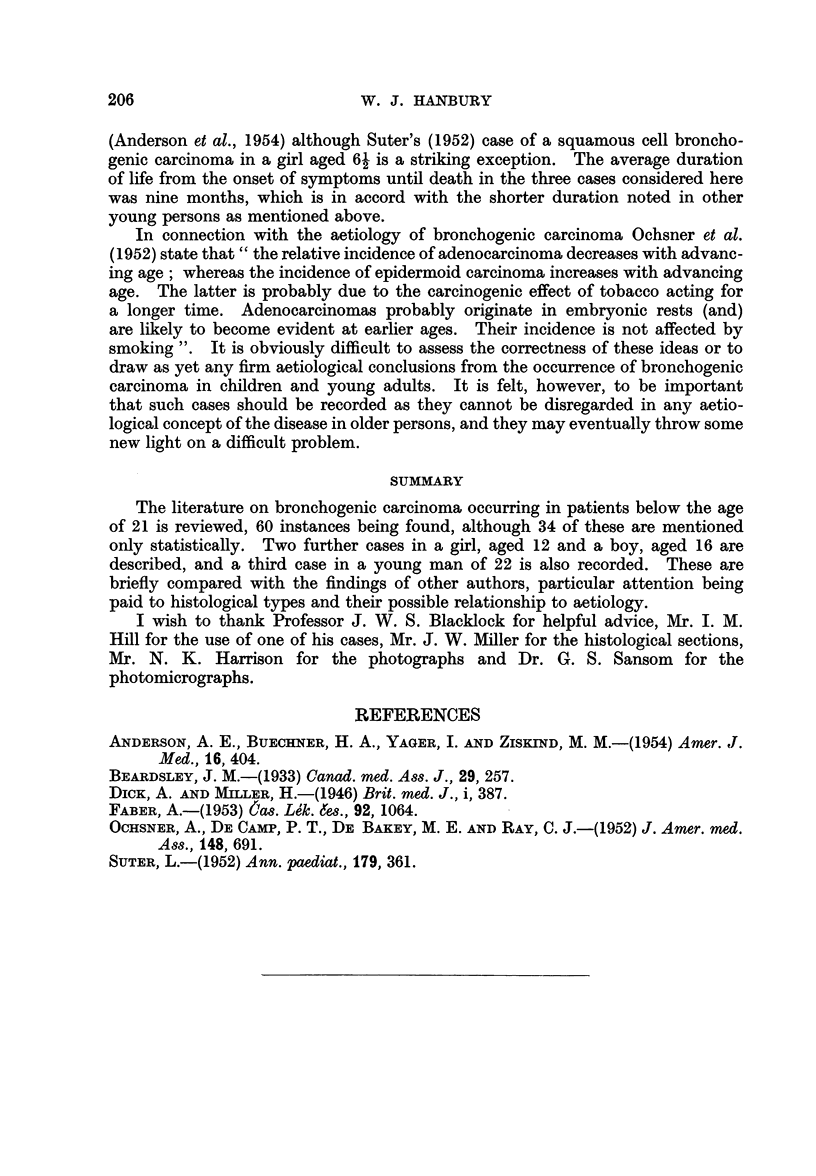# Bronchogenic Carcinoma in Young Persons

**DOI:** 10.1038/bjc.1958.23

**Published:** 1958-06

**Authors:** W. J Hanbury

## Abstract

**Images:**


### BRONCHOGENIC CARCINOMA IN YOUNG PERSONS

W. J. HANBURY

*From the Department of Pathology, St. Bartholomew's Hospital, London, E.C.*1

Received for publication March 29, 1958.

BRONCHOGENIC carcinoma occurring in a group of thirty men less than 40 years of age was the subject of a paper by Anderson *et al.* (1954). It was concluded that the manifestations of the disease in young persons were in marked contrast to those in old people, and it was suggested that the variations represented the effect of different causal mechanisms. In the same paper reports in the literature of bronchogenic carcinoma occurring in children were reviewed, sixteen cases being tabulated. This list was not claimed to be exhaustive, and only those reports which contained adequate histological descriptions were included. It is difficult to determine the exact number of cases reported in the literature, as some are not fully described, while others are of a doubtful nature. However, in addition to the sixteen instances cited by Anderson *et al.* (1954), two cases in children have been reported by Suter (1952) and Faber (1953). Suter mentions five others which are not in Anderson's list, and Beardsley (1933) quotes the cases of Nuscheler, McAldowie and Adler which are also not included. Statistical lists by other authors are referred to by Dick and Miller (1946) and by Suter, accounting for thirty-four additional cases. These figures make a total of sixty instances of bronchogenic carcinoma, occurring below the age of 21, that have been found in the literature, although many of these are mentioned only statistically or not fully described.

The purpose of the present paper is to record two further cases of bronchogenic carcinoma in children, and a third in a young man of 22. The first was the subject of a recent surgical resection, while the other two were found in the Museum of St. Bartholomew's Hospital. In view of the present-day attention focused on the incidence and aetiology of lung cancer, it is considered that these three unusual examples in young persons are of sufficient interest to warrant publication.

### *Case* 1

J. C—, a school-girl aged 12 who lived in outer London, was admitted to hospital with a two weeks' history of left-sided chest pain, which was worse on coughing, her health previously having been good. The only past history connected with the respiratory system was a mild attack of whooping-cough, and there was no family history of chest trouble. X-ray examination revealed a massive opacity in the region of the left lower lobe, and tomograms showed stenosis of the left lower bronchus. A malignant growth was considered possible, especially as the patient was reported to have had a normal chest X-ray four years previously. The Mantoux test (1/1000) was negative, the haemoglobin 80 per cent (Haldane), the white blood count 7800 per cu. mm. and the E.S.R. 71 mm. in 1 hour (Westergren).

A left pneumonectomy was performed and the patient was discharged from hospital five weeks later. Metastases subsequently developed, however, in the liver, and death occurred 9¼ months after the operation (10 months after the onset of symptoms). No post-mortem examination was carried out.

*Pathology of the resected lung*

The specimen consisted of a left lung, the pleura of which was thickened over the lateral aspect of the lower lobe. Sectioning revealed an extensive yellowish-white neoplasm (Fig. 1) measuring 11 × 9 × 8 cm. and appearing to arise from the main lower lobe bronchus distal to the apical branch. The tumour extended laterally to the pleural surface, inferiorly to within 2 cm. of the diaphragmatic surface and medially to project into the hilum of the upper lobe. The cut surface showed areas of tumour necrosis and neoplastic infiltration of broncho-pulmonary lymph nodes. Large blood vessels at the hilum were also surrounded by neoplastic tissue, though no points of rupture could be seen.

Microscopic examination shows an anaplastic carcinoma (Fig. 2) consisting of closely packed round and polyhedral cells with scanty cytoplasm and large vesicular nuclei with prominent nucleoli. Mitoses are numerous and there is evidence of venous, lymphatic (Fig. 3) and alveolar spread. Inflammatory cell infiltrations and areas of necrosis and of lipoid pneumonia are also present. The broncho-pulmonary lymph nodes show direct neoplastic infiltration, but no metastases can be seen in the carinal or other tracheo-bronchial nodes.

*Case 2*

J. C. D—, a boy of 16 from central London, was admitted to hospital (December 4, 1928) complaining of cough and pain in the right side of the chest. He had been well until five months previously when he had been in bed for a week with a cough and "influenza". This was followed a week later by a sudden sharp pain in the right side of the chest, which was worse on taking a deep breath. Although the symptoms of cough and pain improved for short periods, the patient never felt well and was under constant medical treatment, having been diagnosed as a case of rheumatic fever and of pleural effusion at different times. There was no significant family history, and the only past history was the removal of tonsils and adenoids at the age of 10. On examination the boy was pale, but not dyspnoeic or cyanosed, and there were physical signs suggestive of an empyema or lung abscess, but no fluid was obtained on needling the chest. X-ray examination showed a dense opacity of uncertain nature in the lower part of the right lung, and bronchoscopy revealed pus coming from the right main bronchus. There was fairly copious black and yellow sputum which was streaked with blood, but no tubercle bacilli were found. There was also evidence of active chronic sinusitis and pharyngitis. The white blood count fluctuated around 20,000 per cu. mm., and there was irregular pyrexia. The urine was normal.

Exploratory thoracotomy was carried out six weeks after admission, and a thoracoplasty was performed together with drainage of what was thought to be a lung abscess secondary to bronchial obstruction. The pleurae were found to be thickened and adherent, but there was no empyema. Culture of the pus from the lung yielded a growth of Pfeiffer's bacillus. The patient died one month after the operation (7 months after the onset of symptoms) with signs of spreading infection.

*Main post-mortem findings*

*Thoracic contents.*—The right pleural cavity was obliterated and the visceral and parietal layers firmly adherent. Sectioning of the right lung showed a whitish neoplasm (Fig. 4) measuring approximately 6 × 7 × 8 cm. situated around the hilum and extending widely to involve the para-tracheal lymph nodes, the pericardium and part of the wall of the left atrium. The lung tissue peripheral to the tumour showed pneumonic consolidation with pus in the bronchioles. The pericardial sac contained about 7 oz. of thick brownish fluid, and both layers were covered by a shaggy fibrinous exudate (Fig. 4). The left pleura was normal, and the left lung was congested and oedematous, but no metastases were found.

*Liver and kidneys.*—Showed evidence of congestion but were otherwise normal, and no metastases were found. Other organs examined and found to be normal were the brain, thyroid, peritoneum, stomach, intestines, pancreas, mesenteric lymph nodes, suprarenals, ureters and bladder, testes and prostate.

Microscopic examination of the tumour shows a poorly differentiated bronchogenic carcinoma of predominantly oat-cell type (Fig. 5). In places, however, the cells are spindle-shaped or columnar (Fig. 6) and have an alveolar arrangement. There is a well marked fibrous tissue stroma, and areas of haemorrhage and necrosis are fairly numerous. Many small lymphatic vessels are permeated by neoplastic tissue. One of the main bronchi shows extensive acute and chronic inflammatory changes, with hyperplasia of mucous glands and dilatation of the glandular ducts. Many of the smaller bronchi are dilated and contain pus, and the alveoli distal to the tumour contain oedema fluid and inflammatory exudate.

### *Case* 3

W. K—, a male shop-assistant aged 22 from outer London, was admitted to hospital (September 10, 1928) with a history of an unproductive cough during the previous nine months and increasing breathlessness for six months. There had also been occasional pain in the back and in both sides of the chest arteriorly. The patient had a poor appetite, was losing weight, slept badly and had night sweats. A very small clot of blood had occasionally been expectorated. There was a past history of bronchopneumonia and measles as a child, but no significant

---

EXPLANATION OF PLATES

FIG. 1.—Case 1. The left lung sectioned to show the tumour extending from the hilum to the periphery.
FIG. 2.—Case 1. Photomicrograph showing anaplastic carcinoma. Haematoxylin and eosin. × 500.
FIG. 3.—Case 1. Photomicrograph showing carcinomatous infiltration of a dilated pulmonary lymphatic vessel. Haematoxylin and eosin. × 165.
FIG. 4.—Case 2. The right lung sectioned to show the hilar growth extending into adjacent structures. A well-marked fibrinous pericarditis can also be seen.
FIG. 5.—Case 2. Photomicrograph showing a poorly differentiated carcinoma of predominantly oat-cell type. Haematoxylin and eosin. × 530.
FIG. 6.—Case 2. Photomicrograph of the tumour showing an area of spindle-shaped and columnar cells. Haematoxylin and eosin. × 570.
FIG. 7.—Case 3. Photomicrograph of a suprarenal metastasis showing clumps of carcinoma cells with central necrotic areas and a well developed stroma. Haematoxylin and eosin. × 85.
FIG. 8.—Case 3. Photomicrograph showing carcinoma cells of mixed type with a suggestion of glandular arrangement. Haematoxylin and eosin. × 380.

1

2

3

4

Hanbury.

5

6

7

8

Hanbury.

family history. On examination the neck veins were very prominent and there were physical signs of collapse and consolidation of the right lung. Cutaneous nodules were present over the lower part of the sternum and in the lower lumbar region ; a histological section of the latter (now lost) was reported as showing a malignant tumour composed of columnar epithelium having an alveolar arrangement in places. A chest X-ray showed displacement of the heart and trachea towards the right side with an opaque right lung field, suggesting massive collapse of the right lung. Deep X-ray therapy was given, but the patient had increasingly severe attacks of dyspnoea and eventually died 17 days after admission ($9\frac{1}{2}$ months after the onset of symptoms.)

*Main post-mortem findings*

*Lungs.*—A mass of growth was found at the hilum of the right lung. Neoplastic tissue extended laterally into the middle and lower lobes, and medially to involve the lymph nodes at the bifurcation of the trachea ; it was also occluding the right main bronchus and projected for a short distance into the lumen of the left bronchus. Much of the right lung distal to the tumour showed collapse and bronchiectasis, and there were dense pleural adhesions. The left lung showed basal bronchopneumonia.

*Liver.*—Two small subcapsular metastases were present on the inferior surface of the left lobe.

*Kidneys.*—Small subcapsular tumour nodules were present on both sides.

*Suprarenals.*—The right gland was replaced by a mass of firm pinkish-white growth measuring about 8 cm. in diameter. The left gland was spread out over a metastasis of similar appearance measuring about $5 \times 3 \times 2{\cdot}5$ cm.

*Retroperitoneal lymph nodes.*—Some were enlarged by metastatic deposits.

Microscopic examination of the tumour shows a poorly differentiated bronchogenic carcinoma, the cells being arranged in solid clumps and sheets with a well developed stroma and numerous areas of necrosis (Fig. 7). The cells are of mixed spindle-shaped, polyhedral and columnar types, with an occasional papillary glandular arrangement (Fig. 8) and a very occasional suggestion of squamous cell nest formation. Mitoses are fairly numerous.

### DISCUSSION

The observations by Anderson *et al.* (1954), derived from their own cases and from other authors quoted, suggest that in bronchogenic carcinoma in young persons—(1) there is a preponderance of peripheral growths ; (2) there is a relatively small number of the squamous cell variant ; (3) the average duration of life from the onset of symptoms until death is shorter than in older persons.

In considering the three cases reported in this paper, the tumour in the first was too extensive to determine the site of origin, but the last two were certainly hilar growths. The main presenting symptoms were pain in the chest in the first case, cough and pain in the chest in the second, and cough and breathlessness with occasional chest pain and haemoptysis in the third. As regards histological types the tumour in the first case is undifferentiated, in the second of predominantly oat-cell type, while in the third the growth is poorly differentiated but of mixed cellular type. In this connection it is of interest to note the preponderance of undifferentiated and adenocarcinomas in children and young adults

(Anderson *et al.*, 1954) although Suter's (1952) case of a squamous cell bronchogenic carcinoma in a girl aged 6½ is a striking exception. The average duration of life from the onset of symptoms until death in the three cases considered here was nine months, which is in accord with the shorter duration noted in other young persons as mentioned above.

In connection with the aetiology of bronchogenic carcinoma Ochsner *et al.* (1952) state that " the relative incidence of adenocarcinoma decreases with advancing age ; whereas the incidence of epidermoid carcinoma increases with advancing age. The latter is probably due to the carcinogenic effect of tobacco acting for a longer time. Adenocarcinomas probably originate in embryonic rests (and) are likely to become evident at earlier ages. Their incidence is not affected by smoking ". It is obviously difficult to assess the correctness of these ideas or to draw as yet any firm aetiological conclusions from the occurrence of bronchogenic carcinoma in children and young adults. It is felt, however, to be important that such cases should be recorded as they cannot be disregarded in any aetiological concept of the disease in older persons, and they may eventually throw some new light on a difficult problem.

SUMMARY

The literature on bronchogenic carcinoma occurring in patients below the age of 21 is reviewed, 60 instances being found, although 34 of these are mentioned only statistically. Two further cases in a girl, aged 12 and a boy, aged 16 are described, and a third case in a young man of 22 is also recorded. These are briefly compared with the findings of other authors, particular attention being paid to histological types and their possible relationship to aetiology.

I wish to thank Professor J. W. S. Blacklock for helpful advice, Mr. I. M. Hill for the use of one of his cases, Mr. J. W. Miller for the histological sections, Mr. N. K. Harrison for the photographs and Dr. G. S. Sansom for the photomicrographs.

### REFERENCES

ANDERSON, A. E., BUECHNER, H. A., YAGER, I. AND ZISKIND, M. M.—(1954) *Amer. J. Med.*, **16**, 404.
BEARDSLEY, J. M.—(1933) *Canad. med. Ass. J.*, **29**, 257.
DICK, A. AND MILLER, H.—(1946) *Brit. med. J.*, i, 387.
FABER, A.—(1953) *Čas. Lék. čes.*, **92**, 1064.
OCHSNER, A., DE CAMP, P. T., DE BAKEY, M. E. AND RAY, C. J.—(1952) *J. Amer. med. Ass.*, **148**, 691.
SUTER, L.—(1952) *Ann. paediat.*, **179**, 361.